# A Divergent Hepatitis D-Like Agent in Birds

**DOI:** 10.3390/v10120720

**Published:** 2018-12-17

**Authors:** Michelle Wille, Hans J. Netter, Margaret Littlejohn, Lilly Yuen, Mang Shi, John-Sebastian Eden, Marcel Klaassen, Edward C. Holmes, Aeron C. Hurt

**Affiliations:** 1 WHO Collaborating Centre for Reference and Research on Influenza, The Peter Doherty Institute for Infection and Immunity, Melbourne, VIC 3000, Australia; Aeron.Hurt@influenzacentre.org; 2 Molecular Research and Development, Victorian Infectious Diseases Reference Laboratory, Royal Melbourne Hospital at the Peter Doherty Institute for Infection and Immunity, Melbourne, VIC 3000, Australia; Hans.Netter@mh.org.au (H.J.N.); Margaret.Littlejohn@vidrl.org.au (M.L.); Lilly.Yuen@vidrl.org.au (L.Y.); 3 Marie Bashir Institute for Infectious Diseases and Biosecurity, Charles Perkins Centre, School of Life and Environmental Sciences and Sydney Medical School, The University of Sydney, Sydney, NSW 2006, Australia; Mang.Shi@sydney.edu.au (M.S.); js.eden@sydney.edu.au (J.-S.E.); Edward.Holmes@sydney.edu.au (E.C.H.); 4 Centre for Integrative Ecology, Deakin University, Geelong, VIC 3220, Australia; Marcel.Klaassen@deakin.edu.au

**Keywords:** co-evolution, dabbling duck, hepatitis D virus, phylogeny

## Abstract

Hepatitis delta virus (HDV) is currently only found in humans and is a satellite virus that depends on hepatitis B virus (HBV) envelope proteins for assembly, release, and entry. Using meta-transcriptomics, we identified the genome of a novel HDV-like agent in ducks. Sequence analysis revealed secondary structures that were shared with HDV, including self-complementarity and ribozyme features. The predicted viral protein shares 32% amino acid similarity to the small delta antigen of HDV and comprises a divergent phylogenetic lineage. The discovery of an avian HDV-like agent has important implications for the understanding of the origins of HDV and sub-viral agents.

## 1. Introduction

Hepatitis delta virus (HDV) is a human-specific pathogen and the sole member of the genus *Deltavirus*. The HDV genome is unique among known animal viruses but shares similarities with plant sub-viral pathogens named viroids [1,2]. The single stranded, circular RNA genome of HDV is approximately 1700 nucleotides and is therefore the smallest virus infecting mammals. It is ~70% self-complimentary and forms a highly base-paired rod-like structure. It encodes two proteins (small and large delta antigen, S-HDAg, and L-HDAg, respectively) from a single open reading frame. HDV is regarded as a sub-viral pathogen that requires the envelope proteins from the helper hepatitis B virus (HBV) for assembly and release, and subsequently for entry into the host cell [2].

In humans, coinfection with HDV and HBV causes more severe liver disease than is seen in individuals infected with HBV alone, and 15–20 million individuals are estimated to be co-infected with both viruses [3,4]. There are currently eight broad clades, or genotypes, of HDV that have specific geographic distributions [5,6]. Despite the importance of HDV, its origin is unknown and has been the source of debate [6,7,8]. Current hypotheses for the origin of HDV reflect the fact that this virus is currently only found in humans and include: Viroid-like RNA having captured host signalling mRNAs [9], direct origination from the human transcriptome [10], or evolution from a circular host RNA [1,11]. Given the dependence of HDV on the HBV envelope proteins to complete its life cycle and to generate infectious virions it is possible that there been co-evolution between HDV and the helper HBV, although the nature of this relationship has been largely unexplored.

In this study we describe a divergent HDV-like agent found in wild birds and in the absence of detectable duck HBV (DHBV). We demonstrate a number of features shared between HDV and this avian HDV-like agent that are strongly suggestive of common ancestry. This finding has important ramifications for our understanding of both the origin and the co-evolutionary relationships of sub-viral agents and helper viruses.

## 2. Materials and Methods

### 2.1. Ethics Statement

This research was conducted under approval of Deakin University Animal Ethics Committee (permit numbers A113-2010 [6 January 2014] and B37-2013 [17 January 2011]). Banding was performed under Australian Bird Banding Scheme permit (banding authority numbers 2915 and 2703 [04 March 2010]). Research permits were approved by the Department of Environment, Land, Water and Planning Victoria (permit numbers 10006663 [15 February 2013] and 10005726 [20 January 2011]).

### 2.2. Sample Selection, RNA Library Construction and Sequencing

Waterfowl were captured at the Melbourne Water Western Treatment Plant, Victoria, Australia, in 2012 and 2013. Combined oropharangeal and cloacal samples were collected from Grey Teal (*Anas gracilis*), Chestnut Teal (*A. castanea*) and Pacific Black Ducks (*A. superciliosa*). All the birds sampled showed no signs of disease. Fifteen samples were selected, and RNA was extracted using the MagMax *mir*Vana Total RNA isolation Kit (Thermo Scientific, Waltham, MA, USA) and assessed for RNA quality. The ten samples with the highest concentrations were pooled using equal concentrations using the RNeasy MinElute Cleanup Kit (Qiagen, Hilden, Germany). These ten samples comprised six Grey Teals, two Chestnut Teals and four Pacific Black Duck samples. Libraries were constructed and sequenced as per Shi et al. 2016 [12]. Reads have been deposited in the Short Read Archive BioProject PRJNA472212.

### 2.3. RNA Virus Discovery

Contigs were assembled, identified, and abundance calculated as per Shi et al. 2016 [12]. Briefly, sequence reads were demultiplexed and trimmed with Trimmomatic followed by de novo assembly using Trinity [13]. No filtering of host/bacterial reads was performed before assembly. All assembled contigs were compared to the entire non-redundant nucleotide (nt) and protein (nr) database using blastn and Diamond blast [14], respectively, setting an e-value threshold of 1 × 10^−10^ to remove potential false positives. Abundance estimates for all contigs were determined using the RSEM (RNA-Seq by Expectation-Maximization) algorithm implemented in Trinity. All contigs that returned blast hits with paired abundance estimates were filtered to remove all bacterial and host sequences. The virus list was further filtered to remove viruses with invertebrate [12], plant, or bacterial host association using the Virus-Host database (http://www.genome.jp/virushostdb/).

To compare the viral abundance to that found in the host, a blast database was created containing Ribosomal Protein S13 (RPS13) from both Mallard (taxid: 8839) and Chicken (*Gallus gallus*) (taxid: 9031), which has been found to be stably expressed in the Mallard (*Anas playrhynchos*) lower gastrointestinal tract [15].

### 2.4. Characterisation of Novel Hepatitis D-Like Virus

Contigs greater than 1000 bp in length were inspected (Geneious R10). Virus reads were mapped back to the HDV-like contig using the Geneious mapping function to corroborate the contig sequence and to calculate read coverage. Open reading frames were predicted within Geneious and interrogated using the conserved domain database (CDD, https://www.ncbi.nlm.nih.gov/Structure/cdd/wrpsb.cgi), with an expected value threshold of 1 × 10^−5^ We further utilised the HHpred tool [16] available on the MPI Bioinformatics Toolkit platform (https://toolkit.tuebingen.mpg.de/#/tools/hhpred) for additional homology detection and structure prediction. Reference sequences of Hepatitis D representing all eight major clades were downloaded from GenBank. The translation of the HDAg proteins were aligned using MAFFT [17] and gaps trimmed using trimAL (removing gaps that occur in more than 20% of sequences or with a similarity scores lower than 0.005, unless this removes more than 40% of columns) [18]. Maximum likelihood trees were estimated using PhyML 3.0 [19], incorporating the best-fit amino acid substitution model, here JTT + G + F, with 1000 bootstrap replicates using the Montpellier Bioinformatics Platform (http://www.atgc-montpellier.fr/phyml/).

The avian HDV-like agent was subsequently interrogated for conserved features of human HDV. At the genomic level, GC content was calculated within Geneious using a sliding window of ten. To ascertain whether the circular genome folded into a classical unbranched rod structure we utilised a RNA folding algorithm implemented on the Mfold webserver [20]. The identification of the avian HDV-like agent ribozyme was performed in two phases. The TT2NE algorithm was first applied on the genomic and antigenomic ribozyme sequences of the reference HDV genome (GenBank accession X04451.1), and although the models inferred by TT2NE were inaccurate [21], their topologies were distinct and were used as references for screening. The free energy calculated for the HDV genomic and antigenomic ribozyme sequences were −47.35 kcal/mol and −36.70 kcal/mol, respectively. We screened incremental windows of 100 nt starting from the first position of the antigenome of the avian HDV-like agent, including the complementary genomic sequences, with the TT2NE algorithm to locate the potential ribozyme sequences. The incremental size was 50 nt from positions 1 to 500 of the genome sequence and then 10 nt from position 500. All inferred secondary structures that had free energy values similar to the reference models were further evaluated using PseudoViewer [22]. Coiled-coil structures of the ORF were identified using Multicoil [23], and polarity and hydrophobicity calculated within Geneious.

## 3. Results and Discussion

**Figure 1:**
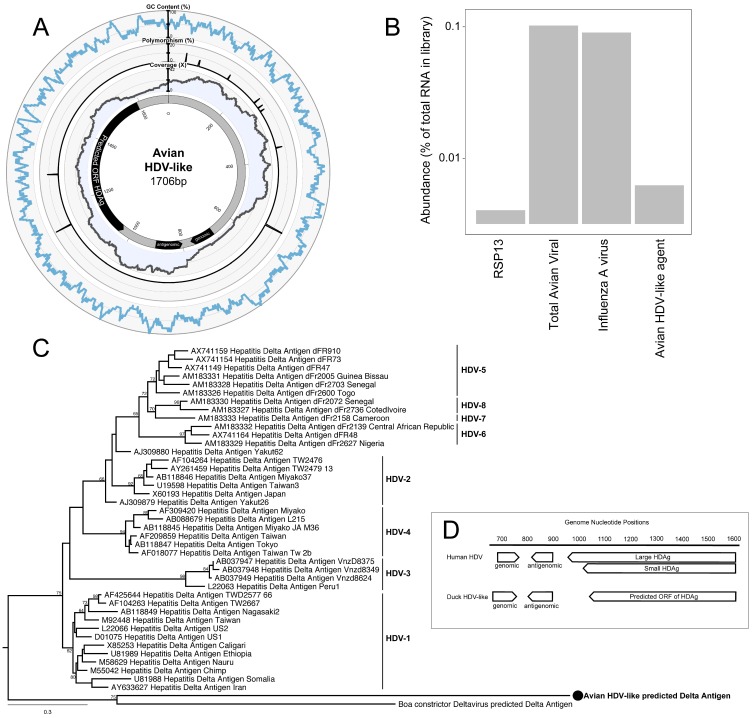
Characteristics of the genome of an avian hepatitis delta virus (HDV)-like agent. (**A**) Avian HDV-like agent genome, annotated with ORFs, genomic, and antigenomic ribozyme sites. Metadata rings include the read coverage, proportion of polymorphisms in reads, followed by GC content. (**B**) Abundance of transcripts in the metatranscriptomic library. Total avian viral abundance was dominated by that of the influenza A virus. However, the abundance of HDV is higher than that of Ribosomal protein S13 (RPS13), a stably expressed reference gene in Mallards (*Anas platyrhynchos*). (**C**) Maximum likelihood phylogeny of the HDAg protein. Representative human HDAg sequences fall into the currently described clades HDV1-8 (5). The scale bar represents the number of amino acid substitutions per site. The phylogeny is rooted between the human and avian/snake viruses. (**D**) Location of genomic and antigenomic ribozyme sequences, and the predicted ORF of the delta antigen in the avian HDV-like genome compared to their location in the HDV genome sequence (GenBank accession X04451.1).

As part of an avian meta-transcriptomic study, we identified a genome related to that of HDV, indicating that a novel and divergent HDV-like agent is present in the bird population. RNA sequencing of the rRNA depleted library resulted in 20,945,917 paired reads, which were assembled into 163,473 contigs, 279 of which were most similar to virus sequences in the GenBank non-redundant protein database (nr). Avian viral transcripts were highly abundant in the library, largely representing influenza A virus. RNA from an avian HDV-like agent was ten-fold less abundant than that of influenza A virus, but was more abundant than RNA from the host reference gene RPS13 that is stably expressed in ducks [15] (Figure 1B). Notably, exogenous DHBVwas not identified in this meta-transcriptomic library.

The full genome of the novel avian HDV-like agent was represented by a single contig (GenBank accession MH824555), wherein the virus genome was duplicated, the result of sequencing a circular genome of 1706 nucleotides (Figure 1A). Unlike HDV which has a GC content of around 60% [24], the GC content of the avian HDV-like agent is only 51%, with no significant peaks or troughs of GC content anywhere in the genome (Figure 1A). The predicted avian HDV-like protein (avHDAg) shares 32.2% amino acid similarity to characterized HDAg proteins in the Genbank nr database. A conserved domain search identified the ORF in the virus genome as clearly representing the HDAg (e-value 3.40 × 10^−10^). We further used the HHpred tool to detect protein homology, and the ORF matched the HDAg (e-value 1.22 × 10^−22^) with a probability of 99.79%. Critically, the conserved domain search, HHpred search, and the blast databases contain the cellular homolog of CCDC85B (previously referred to as DIPA [8]), and the predicted avDHAg did not match this homolog. Phylogenetic analysis indicated that avHDAg was highly divergent from all HDAgs encoded by known human HDV genotypes, falling as a divergent sequence in a similar manner to that of a recently described delta-virus identified in a Boa constrictor (*Boa constrictor sabogae*) [25] (Figure 1C).

**Figure 2:**
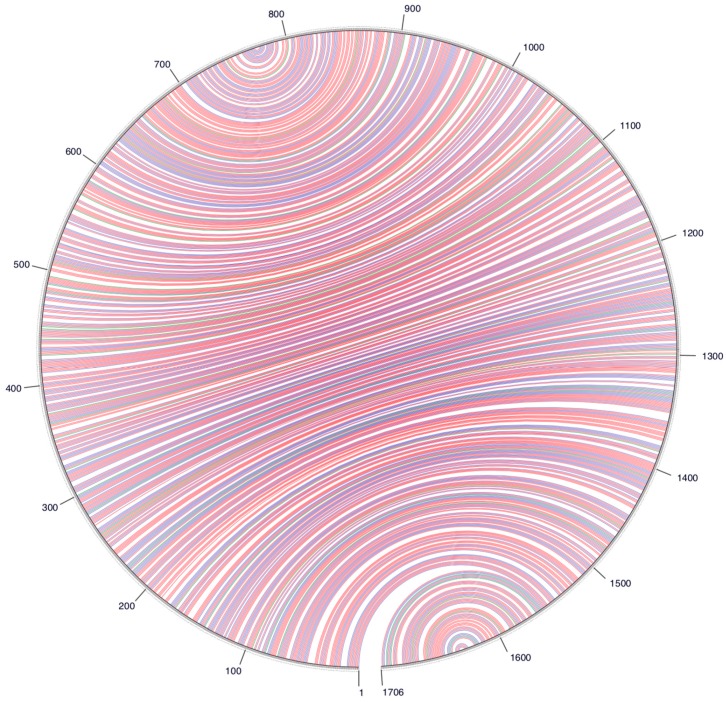
A circle graph showing the base pairing of the circular RNA genome structure of the avian HDV-like agent into an unbranched rod-like structure. The circle circumference represents the genome sequence, and the arcs represent the base pairing. Colouring of arcs: Red for G-C pairing, blue for A-U pairing, green for G-U pairing, and yellow for other types of pairings.

**Figure 3:**
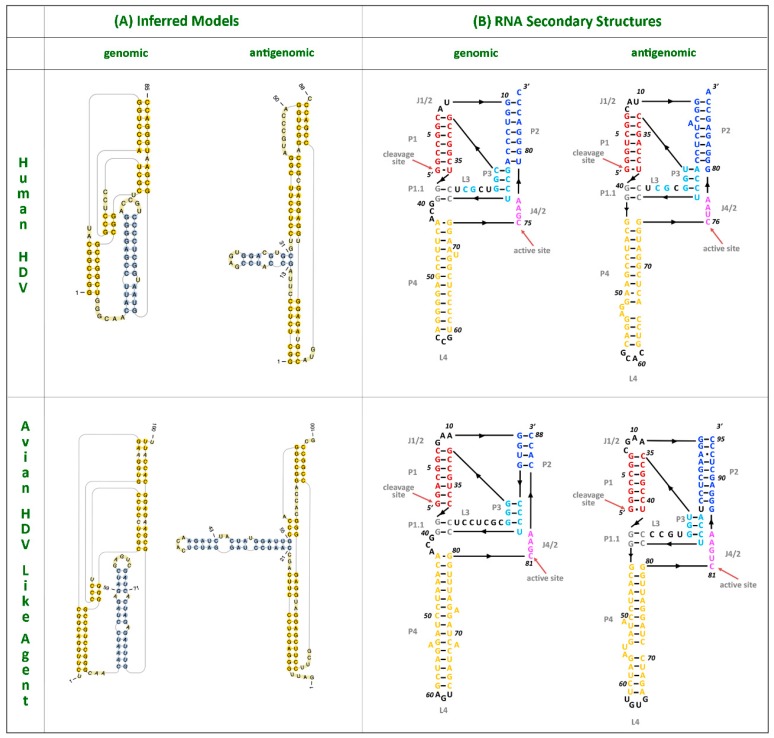
HDV ribozymes. (**A**) Secondary structures of the genomic and antigenomic ribozymes inferred using the TT2NE algorithm. The HDV ribozyme models were used as reference to screen for the ribozyme sequences in the avian HDV-like genome sequence. (**B**) Re-drawn secondary structures of the genomic and antigenomic ribozymes based on the secondary structures shown in the review by Webb and Luptak (21).

Despite this divergence, the avian HDV-like agent and HDV shared many features that are strongly suggestive of common ancestry. In accordance with the unbranched, rod-like genome structure described for HDV, we demonstrate that the predicted circular RNA genome of the HDV-like agent also folded into a classic unbranched rod-like structure (Figure 2). Importantly, consistent with HDV, the genome of the avian HDV-like agent had the capacity to express a protein, avHDAg, and contained sequences reminiscent of the HDV genomic and antigenomic ribozymes [26], as well as the HDV-like ribozymes [21]. To be consistent with the HDV nomenclature, we regard the sequence with the avHDAg ORF as the antigenome. Given that the antigenomic HDV ribozyme is located approximately 100 nucleotides downstream of the small HDAg ORF in the 3′-direction, we examined the corresponding antigenomic region of the avian HDV-like agent for the presence of ribozyme like sequences (avHDAg ORF between nucleotides 1033 and 1590, Figure 1A). Two segments in the avian HDV-like genome were identified as potential genomic and antigenomic ribozyme sequences. Their sizes and locations were very similar to the ribozymes in the reference HDV genome sequence (Accession X04451.1) (Figure 1D and Figure 3). The potential genomic and antigenomic ribozyme sequences were approximately 88 and 95 nt in length, respectively (compared to 85 and 89 nt of the human HDV ribozyme sequences [26]), and the calculated free energies were −40.53 kcal/mol and −38.68 kcal/mol, respectively. When the inferred structures were re-drawn based on the canonical secondary structures of the human HDV ribozymes [21], both potential genomic and antigenomic duck ribozyme sequences displayed the ability to be folded into classic HDV ribozyme secondary structures (Figure 3). This includes the five paired (P) segments forming two coaxial stacks (P1 stacks on P1.1 and P4, while P2 stacks on P3), with these two stacks linked by single-stranded joining (J) strands J1/2 and J4/2, as described by [21].

**Figure 4:**
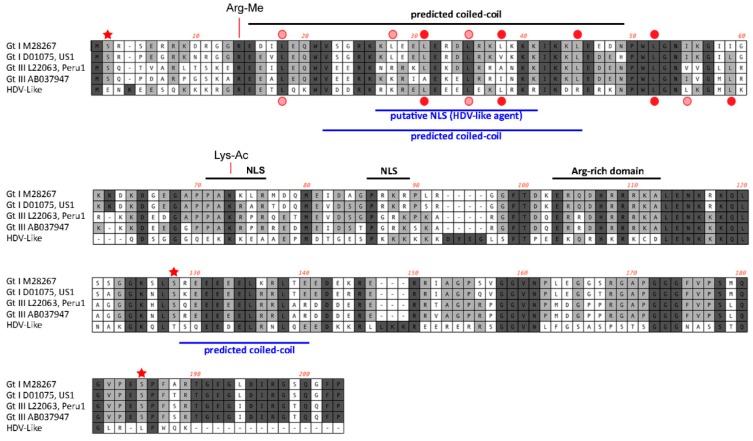
Features of the predicted HDAg protein. Alignment of the amino acid sequences (small delta antigen) translated from the genomes of HDV and the avian HDV delta-like agent. The potential coiled-coil region is highlighted, including the presence of leucine residues in the correct spacing for a leucine zipper (filled red circle). The delta antigen does not have a strict requirement for leucine in the d-position of the heptad repeat. Additional leucine residues are shown by circles in light red. Serine residues that are conserved between different HDV genotypes and post-translationally modified (phosphorylated) are highlighted with an asterisk. The conserved arginine and lysine residues modified by methylation (Arg-Me) and acetylation (Lys-Ac) are indicated. NLS: Nuclear localisation signal.

The avian HDV-like genome contains a predicted ORF for the avHDAg that encodes 185 amino acids. The first AUG is located within a Kozak consensus sequence with a G in the +4 position, and Adenine in position −3 [27]. Downstream of the coding sequence after 105 nucleotides, the RNA genome encodes signals, which are typical for the 3′-end of eukaryotic mRNAs required for adding the poly(A) tail, the highly conserved 5′-AAUAAA recognition sequence followed by the 5′-CA-3′ cleavage sequence after 14 nucleotides [28,29]. In contrast to HDV, the genome of the avian HDV-like agent does not contain an editing site as identified for HDV. For HDV, the editing event converts the “UAG” stop codon into the tryptophan “UGG” codon [30], which extends the reading frame by an additional 18 amino acids, resulting in the synthesis of the L-HDAg. However, the avian HDV-like genome potentially provides additional reading frames by +1 and +2 frame shifts extending the ORF of 185 amino acids by 18 and 65 amino acids, respectively. The nucleotide sequence encoding the additional 65 amino acids overlaps with the poly (A) signal sequence, and is therefore less likely to be translated from a functional mRNA molecule. The C-terminal region of the 185 amino acid protein, including the potential frame-shifted extensions do not contain a C-X-X-X isoprenylation site, which is required for the synthesis of a functional L-HDAg to support HDV assembly and release [31]. Consistent with the presence of a coiled-coil domain in the N-terminal region of the HDAg, the protein avHDAg also contains a coiled-coil domain predicted in the N-terminal region between amino acids 22 and 44, probably facilitating dimerization (Figure 4A) [32,33]. The delta antigen can undergo a variety of posttranslational modifications, which can modulate biological function [34,35]. Serine residues identified as phosphorylation sites are not conserved in the avHDAg; however, an arginine at position 14 (R13 in delta-antigen) or lysine at position 73 (K72 in delta antigen), which are targets for methylation and acetylation, respectively, are conserved (Figure 1). Due to the substantial differences between the HDAg and the avHDAg, post-translation modifications will have to be re-evaluated for the avHDAg. The isoelectric point (pI value) is similar between avHDAg (estimated pI = 10.4) and S-HDAg (estimated pI = 10.3 to 10.8, depending on the HDV isolate).

The peptide has an approximately equal number of hydrophobic, acidic, basic, and neutral amino acids. Although the distribution is similar to human HDV (Genbank accession X04451.1), the latter has a larger proportion of hydrophobic residues (up to 31%). Both HDV and the avian HDV-like agent have a long stretch of neutral and hydrophobic residues towards the C-terminal, and the increased hydrophobicity of human HDV may be attributed to a longer peptide and hydrophobic region (Figure 4).

## 4. Conclusions

There are a number of hypotheses for the origin of HDV, including that viroid-like RNA captured host signalling mRNAs [9], that HDV originated directly from the human transcriptome [10], or evolved from a circular host RNA found in hepatocytes that was able to replicate [1,11]. A central component of these hypotheses is that HDV exists only in humans. However, the discovery of a related HDV-like genome in birds and snakes [25] with distinct similarities to the HDV genome, including self-complementarity and ribozyme folding, as well as clear differences (no ORF extension in the same frame downstream of the stop codon), suggests a divergent evolutionary pathway of HDV and HDV-like pathogens. The lack of DHBV in the metagenomics library is notable, and may be due to a number of factors such as: (a) DHBV is primarily a liver infection and therefore oropharyngeal/cloacal swabs are not the optimal sample for DHBV detection; (b) DHBV may have been suppressed by the avHDV-like agent, in the same way that HBV replication is inhibited by HDV, resulting in low HDBV viral load and a lack of detection by metagenomics; or (c) the avHDV-like agent does not depend on a hepadnavirus for the completion of its replication cycle. Hetzel et al., (2018) similarly reported the absence of hepadnavirus and also demonstrated the active replication of a snake HDV-like agent. This suggests that non-human HDV-like agents may use other viruses to obtain a lipid envelope to make infectious particles, which clearly merits additional study. As such, the discovery of the genome of an avian HDV-like agent has important implications for our understanding of both the origin and the co-evolutionary relationships of the sub-viral agents with helper viruses, including the dependence of HDV on the HBV envelope protein.

## Supplementary Materials

Supplementary File 1
