# Supplementary material for: A Divergent Hepatitis D-Like Agent in Birds
Source: Viruses. 2018 Dec 17;10(12):720. doi: 10.3390/v10120720 (PMC6315422; doi:10.3390/v10120720)
Supplement: Supplementary File 1 [file viruses-10-00720-s001.pdf]

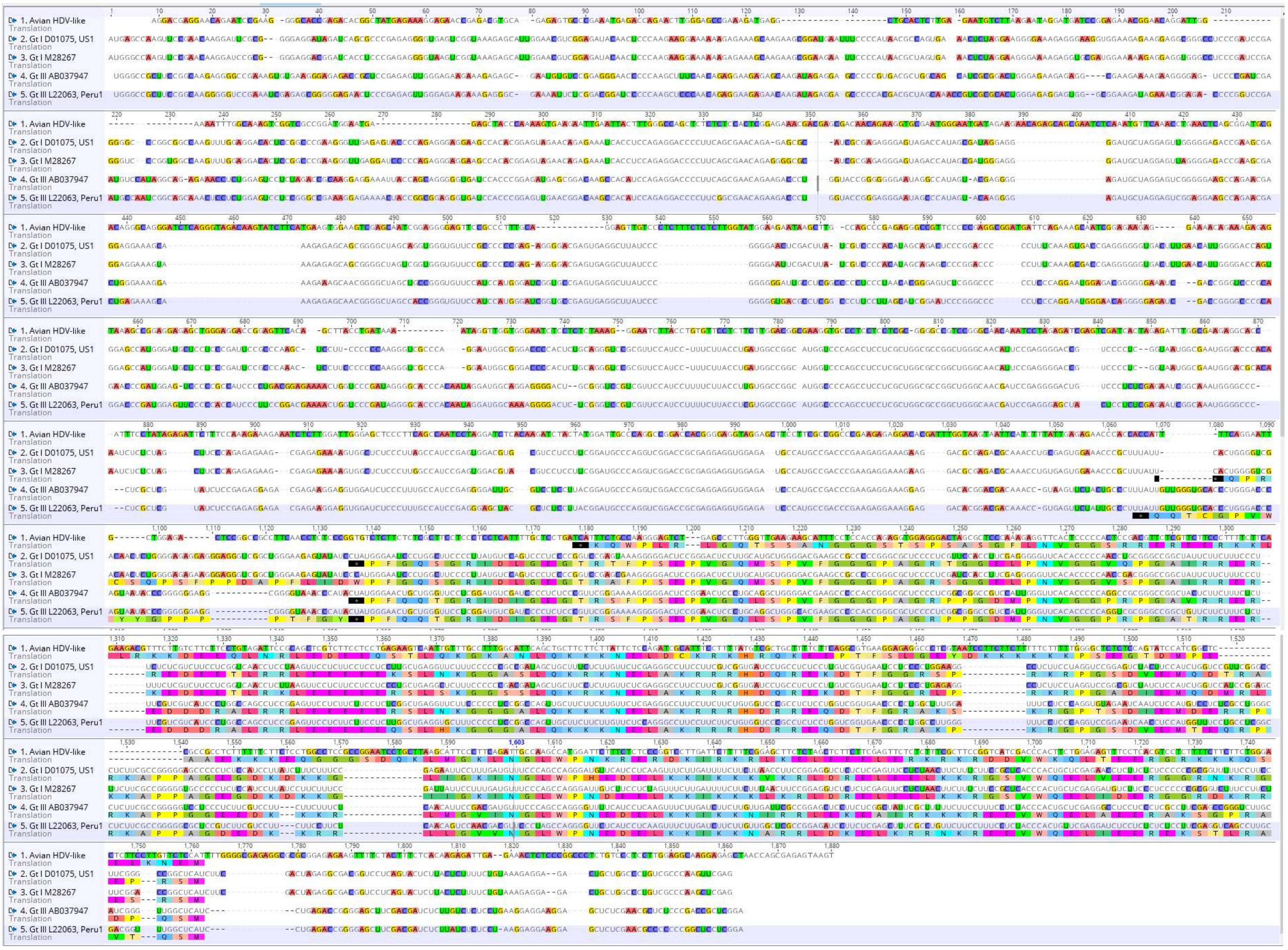

Supplemental Nucleotide Alignment. Nucleotide alignment using the MAFFT algorithm of the avian HDV-like virus and reference HDV viruses. The translation of the HDAg and predicted avHDAg is also shown, demonstrating that despite nucleotide divergence, there is conservation at the amino acid level. The amino acid alignment is presented in Fig 4.
